# RNA shotgun metagenomic sequencing of northern California (USA) mosquitoes uncovers viruses, bacteria, and fungi

**DOI:** 10.3389/fmicb.2015.00185

**Published:** 2015-03-24

**Authors:** James Angus Chandler, Rachel M. Liu, Shannon N. Bennett

**Affiliations:** Department of Microbiology, California Academy of SciencesSan Francisco, CA, USA

**Keywords:** metagenomics, shotgun sequencing, microbiota, *Culex pipiens*, *Culiseta*, *Ochlerotatus*, *Bunyaviridae*, *Rhabdoviridae*

## Abstract

Mosquitoes, most often recognized for the microbial agents of disease they may carry, harbor diverse microbial communities that include viruses, bacteria, and fungi, collectively called the microbiota. The composition of the microbiota can directly and indirectly affect disease transmission through microbial interactions that could be revealed by its characterization in natural populations of mosquitoes. Furthermore, the use of shotgun metagenomic sequencing (SMS) approaches could allow the discovery of unknown members of the microbiota. In this study, we use RNA SMS to characterize the microbiota of seven individual mosquitoes (species include *Culex pipiens, Culiseta incidens,* and *Ochlerotatus sierrensis*) collected from a variety of habitats in California, USA. Sequencing was performed on the Illumina HiSeq platform and the resulting sequences were quality-checked and assembled into contigs using the A5 pipeline. Sequences related to single stranded RNA viruses of the *Bunyaviridae* and *Rhabdoviridae* were uncovered, along with an unclassified genus of double-stranded RNA viruses. Phylogenetic analysis finds that in all three cases, the closest relatives of the identified viral sequences are other mosquito-associated viruses, suggesting widespread host-group specificity among disparate viral taxa. Interestingly, we identified a *Narnavirus* of fungi, also reported elsewhere in mosquitoes, that potentially demonstrates a nested host-parasite association between virus, fungi, and mosquito. Sequences related to 8 bacterial families and 13 fungal families were found across the seven samples. *Bacillus* and *Escherichia/Shigella* were identified in all samples and *Wolbachia* was identified in all *Cx. pipiens* samples, while no single fungal genus was found in more than two samples. This study exemplifies the utility of RNA SMS in the characterization of the natural microbiota of mosquitoes and, in particular, the value of identifying all microbes associated with a specific host.

## Introduction

The diversity of animal-associated microbes, collectively called the microbiota, and their ubiquitous role in host ecology, physiology, and evolution has reached new levels of appreciation with the advent of next-generation sequencing technologies (Foster et al., 2012; McFall-Ngai et al., 2013).

Culture-dependent methods are limited in their capacity to capture a full picture of the microorganisms present, as only a minority of microbes are easily culturable under “standard” laboratory conditions (Amann et al., 1995 but see Donachie et al., 2007). Metagenomic PCR circumvents this issue by identifying organisms directly from environmentally acquired nucleic acids, often using taxon-specific primers that target genes such as the ribosomal small subunit (Schmidt et al., 1991; Handelsman et al., 1998). However, this method has known issues related to primer selection, chimera formation, and gene copy number (Ashelford et al., 2006; Kembel et al., 2012; Ghyselinck et al., 2013; Klindworth et al., 2013). Shotgun metagenomic sequencing (SMS), which does not rely upon an initial PCR step, can avoid the limitations of culture-dependent and PCR-based methods (Venter et al., 2004; Haas et al., 2011). Combined with high throughput sequencing technologies, SMS has been successfully used to characterize the microbiota of honeybees (Runckel et al., 2011; Engel et al., 2012), termites (Warnecke et al., 2007), humans (Qin et al., 2010), and a variety of mammals (Muegge et al., 2011). SMS has an added benefit that the data produced is not restricted to a single taxon (i.e., only bacteria or only fungi) and has been used to identify a variety of microorganisms associated with a single host (Runckel et al., 2011). In this study, we use SMS and high throughput sequencing to examine the microbiota of mosquitoes collected in northern California, USA.

Mosquitoes are vectors of many clinically and economically important diseases, such as malaria and dengue (Morens et al., 2004; Fukuda et al., 2011). Additionally, mosquitoes carry many non-pathogenic microbes (Minard et al., 2013) and some of these are prime candidates for symbiont mediated transmission disruption. For example, the intracellular bacterium *Wolbachia* can affect dengue transmission in *Aedes aegypti* (Walker et al., 2011) and reduces the titer of *West Nile virus* in *Culex quinquefasciatus* (Glaser and Meola, 2010). Genetically modifying bacteria that are naturally associated with mosquitoes is also a promising approach to transmission disruption, as has been shown with *Anopheles* mosquitoes and malaria (Wang et al., 2012). These studies and others (reviewed in Cirimotich et al., 2011) were made possible through a previous understanding of the mosquito microbiota.

Numerous culture-dependent and culture-independent studies have examined the bacterial communities associated with mosquitoes (reviewed in Minard et al., 2013). Generally, the bacterial taxa Gammaproteobacteria and Firmicutes are major components of the mosquito microbiota, but other groups, such as the Alphaproteobacteria and the Betaproteobacteria, are also present (Minard et al., 2013). Studies investigating the mosquito microbiota have shown that its composition is based on both environmental (such as habitat) and host-intrinsic (such as sex, species, and developmental stage) factors (Minard et al., 2013). The function of the microbiota remains unclear in many cases, although its experimental removal does arrest larval development in three different mosquito species (Coon et al., 2014).

As with other animal hosts [e.g., humans (Huffnagle and Noverr, 2013) and *Drosophila* (Broderick and Lemaitre, 2012)], fungi associated with mosquitoes are relatively understudied compared to their bacterial counterparts. Most previous work investigating the fungi associated with mosquitoes has focused on entomopathogenic fungi and their use in mosquito control (Scholte et al., 2004; de Faria and Wraight, 2007). However, to our knowledge, there are no studies that have used culture-independent techniques to characterize the total fungal communities of natural populations of mosquitoes.

Shotgun metagenomic sequencing has previously been used to investigate the mosquito microbiota. In particular, the viral diversity associated with wild-caught mosquitoes has been characterized and these studies have shown that SMS is sufficient to recover and identify viruses from a variety of taxonomic groups (Ma et al., 2011; Ng et al., 2011; Cook et al., 2013; Chandler et al., 2014). Furthermore, mosquitoes that have been laboratory infected with known mosquito-vectored viruses (such as dengue, yellow fever, or chikungunya) have been subjected to SMS (Bishop-Lilly et al., 2010; Hall-Mendelin et al., 2013). In addition to successful identification of the viruses and mosquitoes, genetic material from both bacteria and fungi were recovered via SMS.

Lab-infected mosquito studies (Bishop-Lilly et al., 2010; Hall-Mendelin et al., 2013) suggest the feasibility of using SMS to identify both viruses and other microorganisms (such as bacteria and fungi) in wild populations of mosquitoes, however, to our knowledge, this has never been done. Because symbiont mediated transmission disruption is an emerging tool in controlling vector-borne diseases (reviewed in Cirimotich et al., 2011; Weiss and Aksoy, 2011), SMS of wild mosquitoes may be particularly informative because it simultaneously identifies both viruses and other symbionts and could uncover any correlation between the two in the same host. This study represents a proof of concept in characterizing microbiota using a taxonomically broad approach that could ultimately prove useful in exposing significant novel interactions between microbes. In particular, we use SMS to characterize the microbiota of seven individual mosquitoes of three different species that were collected from a variety of natural habitats in northern California, USA, which is an area where *West Nile virus* has been reported. Using RNA-based SMS on the Illumina platform, we identify sequences related to viruses, bacteria, and fungi in each individual. Furthermore, we were able to verify mosquito species identities using SMS data alone. This work exemplifies the utility of SMS to study the natural microbiota of mosquitoes and we hope it prompts future research in this area.

## Materials and Methods

### Collection and Identification

**FIGURE 1:**
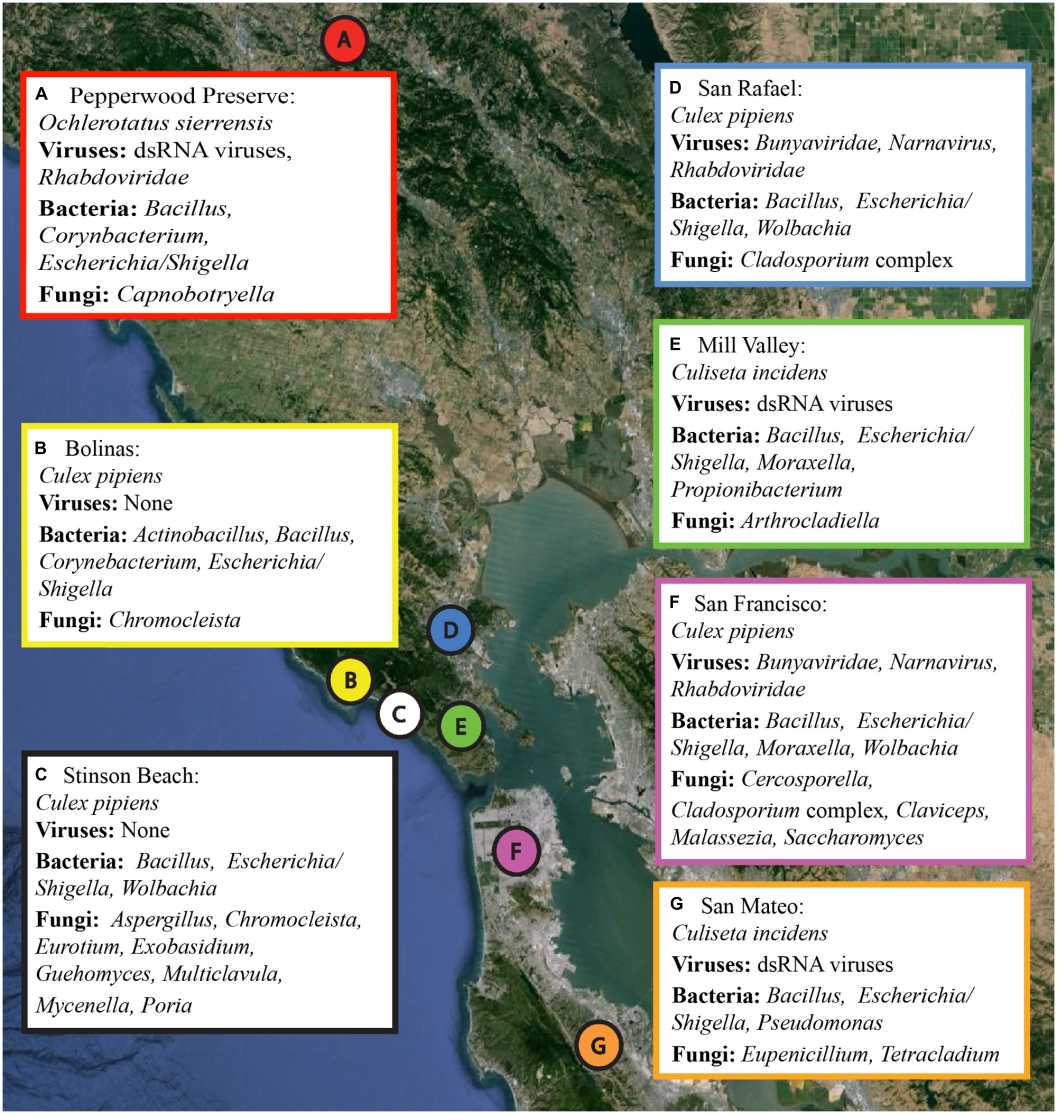
**Collection locations and a summary of the microbial sequences identified in each sample.** Locations A through G correspond to the samples as listed in **Table 1**. A summary of the viral, bacterial, and fungal sequences identified in each sample is included. Details for each microbial group can be found in **Tables 2–4**. The map was created using Google Earth Pro, with data from the U.S. Navy, LDEO-Columbia, NSF, NOAA, SIO, NGA, MBARI, and GEBCO.

Several hundred mosquitoes were collected from seven locations in northern California (Pepperwood Preserve, Bolinas, Stinson Beach, San Rafael, Mill Valley, San Francisco, and San Mateo) during March–November 2013 (**Figure 1**). Locations were chosen to represent a range of habitats, from sylvatic/wild (e.g., Pepperwood Preserve) to urban (e.g., San Francisco), (**Table 1**). Collections occurred under the permit and permission agreements of the Marin/Sonoma Mosquito and Vector Control District, or on private lands with the owners’ permission.

**Table 1:** Sample details.

Map	Location	Habitat	Species	Library name	Total reads	Number of reads after quality control	Number of contigs
A	Pepperwood Preserve	Sylvatic/wild	*Ochlerotatus sierrensis*	pepp.ochl	35,807,449	32,066,403	29,911
B	Bolinas	Rural	*Culex pipiens*	boli.cpip	32,644,864	29,446,138	30,686
C	Stinson Beach	Rural	*Culex pipiens*	stin.cpip	31,123,780	27,519,860	29,234
D	San Rafael	Urban	*Culex pipiens*	sraf.cpip	36,917,070	32,872,651	44,558
E	Mill Valley	Rural/suburban	*Culiseta incidens*	mill.culi	36,248,881	32,552,420	13,577
F	San Francisco	Urban	*Culex pipiens*	sfra.cpip	33,906,608	30,669,364	53,542
G	San Mateo	Suburban/urban	*Culiseta incidens*	smat.culi	35,641,996	32,203,598	110,242

We employed several methods to collect mosquitoes, including hand nets, gravid traps baited with hay-infused water, or Zumba^TM^ traps baited with carbon dioxide gas (CO_2_), heat packs, and SkinLure^TM^. All samples were frozen within 24–48 h of being trapped and stored at -80°C. We identified mosquitoes morphologically using a dissecting microscope and key (Bohart and Washino, 1978), retaining voucher specimens for each species and trapping event. In several cases, morphological identification was verified by sequence identity for cytochrome oxidase I (COI) using a leg from the voucher specimen.

### DNA Extraction, Library Preparation, and Sequencing

From the several hundred mosquitoes collected, one representative individual female from each of the seven different collection sites was selected for SMS sequencing (**Table 1**). Prior to processing, whole mosquitoes were washed in 70% ethanol, distilled water, and phosphate-buffered saline (PBS) solution to remove external microbes. While we did not explicitly test the final wash for complete removal of external microbes, a similar wash protocol for *Drosophila* was found to be sufficient for this purpose (Chandler et al., 2011). Washed samples were then individually homogenized in PBS with steel Lysing Matrix I beads (MP Biomedicals).

Samples were prepared for sequencing by first undergoing total RNA isolation, rRNA subtraction, reverse transcription, and random amplification into cDNA libraries. For RNA extraction, we used the MasterPure^TM^ Complete DNA and RNA Purification kit according to the manufacturer’s protocols. After DNase treatment, pellets were resuspended in 30 μl of TE buffer and 1 μl of RiboGuard^TM^ was added to each tube. Next, we performed ribosomal subtraction using Ribo-Zero^TM^ Gold (magnetic beads, human/rat/mouse kit). While we did not design our experiment to explicitly test the efficacy of Ribo-Zero^TM^ Gold on the removal of mosquito ribosomal RNA (rRNA), we note that the final percentage of mosquito 28S and 18S rRNA per library was, on average, 29.5% and 2.5%, respectively (data not shown). This was determined using the default settings of Bowtie 2 (Langmead et al., 2009) to map the quality checked reads (see below) to any contigs with a closest match to insect large subunit (LSU) and small subunit (SSU) rRNA.

Samples then underwent random reverse transcription followed by random amplification. Samples were tagged with a molecular ID tag (MID) and pooled equimolar for a single flow cell of sequencing. Sequencing was done on the Illumina HiSeq 2000 at HudsonAlpha Institute for Biotechnology, using an insert size of 100 bp paired-end reads. No water or blank sample was included as a negative control [although this is recommended for future sequencing runs (Salter et al., 2014)]. Raw, unprocessed sequencing reads are available through the NCBI Short Read Database as part of BioProject PRJNA269777.

### Sequence Processing and Taxonomy Assignment

An average of 35 million reads were produced per library (details for each library are available in **Table 1**). Sequences were quality checked and assembled using the A5 pipeline with the metagenome flag (Tritt et al., 2012). A5 combines sequence quality control, adapter trimming, and contig assembly. The SGA software package removes low quality reads and corrects sequencing errors ^1^ and Tagdust removes sequencing adapter contamination (Lassmann et al., 2009). Cleaned sequences are used to build contigs with the IDBA-UD assembler (Peng et al., 2012).

To identify any viral sequences associated with these mosquitoes, contigs were translated into all six frames and any open reading frames (ORFs) longer than 100 amino acids in length were further examined. These ORFs were queried against a custom blast database using the blastp algorithm. We used an *e*-value cutoff of 1 × 10^-3^, which is approximately equivalent, for our database, to 30% similarity over 100 amino acids and 20% similarity over 500 amino acids. This custom database contains the entire NCBI non-redundant protein database, the PhAnToMe phage protein database (Aziz et al., 2012), the NCBI Viral RefSeq Database, and the *Aedes aegypti*, *Anopheles gambiae*, and *Culex quinquefasciatus* protein databases (Megy et al., 2012). The rationale for combining databases was to ensure that viral and phage proteins were present, while simultaneously reducing the false positive rate by the inclusion of all possible non-viral or non-phage proteins. While none of the mosquitoes examined in this study have their complete genomes sequenced, we note that this would not reduce our ability to detect viral sequences; rather this simply increases the false positive rate. All ORFs with a closest match to “virus” or “phage” were manually queried to the NCBI website to confirm their identity. Confirmed viral sequences are available on NCBI through the GenBank accession numbers KP642114 to KP642128.

To identify any bacterial and fungal sequences in the datasets, the non-translated contigs were queried using the blastn algorithm (Altschul et al., 1990) to the SILVA SSU and LSU Reference Databases (Release 111; Quast et al., 2013). Contigs with a closest match to “Bacteria” and “Fungi” when queried to the SSU and LSU databases, respectively, and were longer than the 300 bp, were then submitted to Ribosomal Database Project’s (RDP) Classifier for taxonomic assignment (Wang et al., 2007). Contigs above a 90% genus (SSU) or 70% family (LSU) confidence cutoff were considered reliable hits (**Table 3 and 4**). All intermediate files, include blast results and fasta files of significant hits are available at .

To identify the mosquito host taxa, the non-translated contigs were queried to a custom-made database of mosquito COI genes. Matches were manually queried to the NCBI website to confirm their identity. Mosquito COI sequences are available on NCBI through the GenBank accession numbers KP293419 to KP293425.

### Viral Phylogenetic Analysis and Coverage Estimation

**FIGURE 2:**
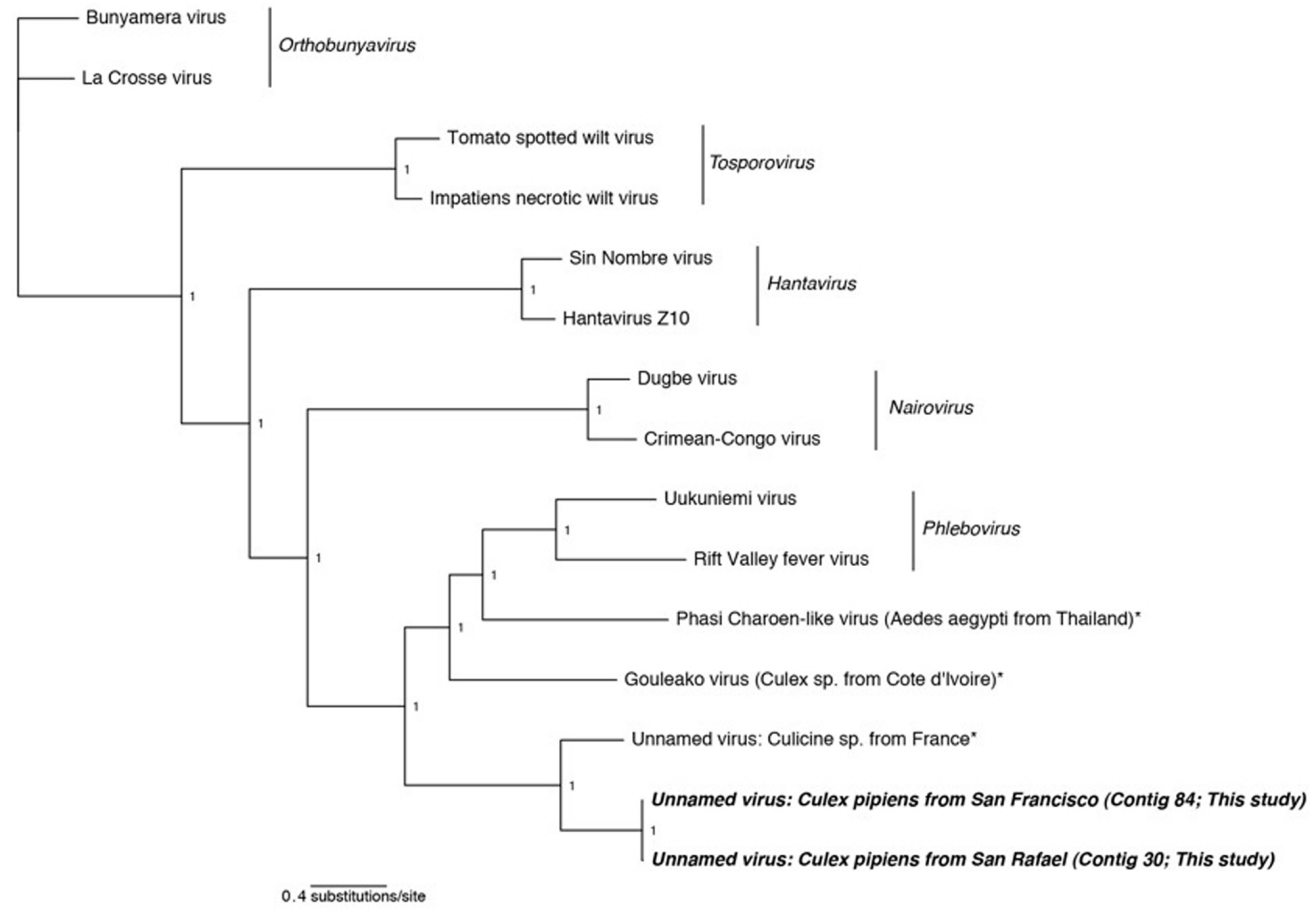
**Phylogenetic history of all genera in the *Bunyaviridae* including sequences from this study.** This consensus phylogeny was generated in MrBayes (Ronquist and Huelsenbeck, 2003) based on two independent chains run for one million generations to convergence. The average standard deviation of split frequencies was 0.000008. Posterior node probabilities are shown at nodes. Branch lengths are scaled to substitutions/site. Viral sequences uncovered in this study are labeled as such. Closely related mosquito-associated viruses are marked with an asterisk. The accession numbers of all sequences are available at http://dx.doi.org/10.6084/m9.figshare.1247641

**FIGURE 3:**
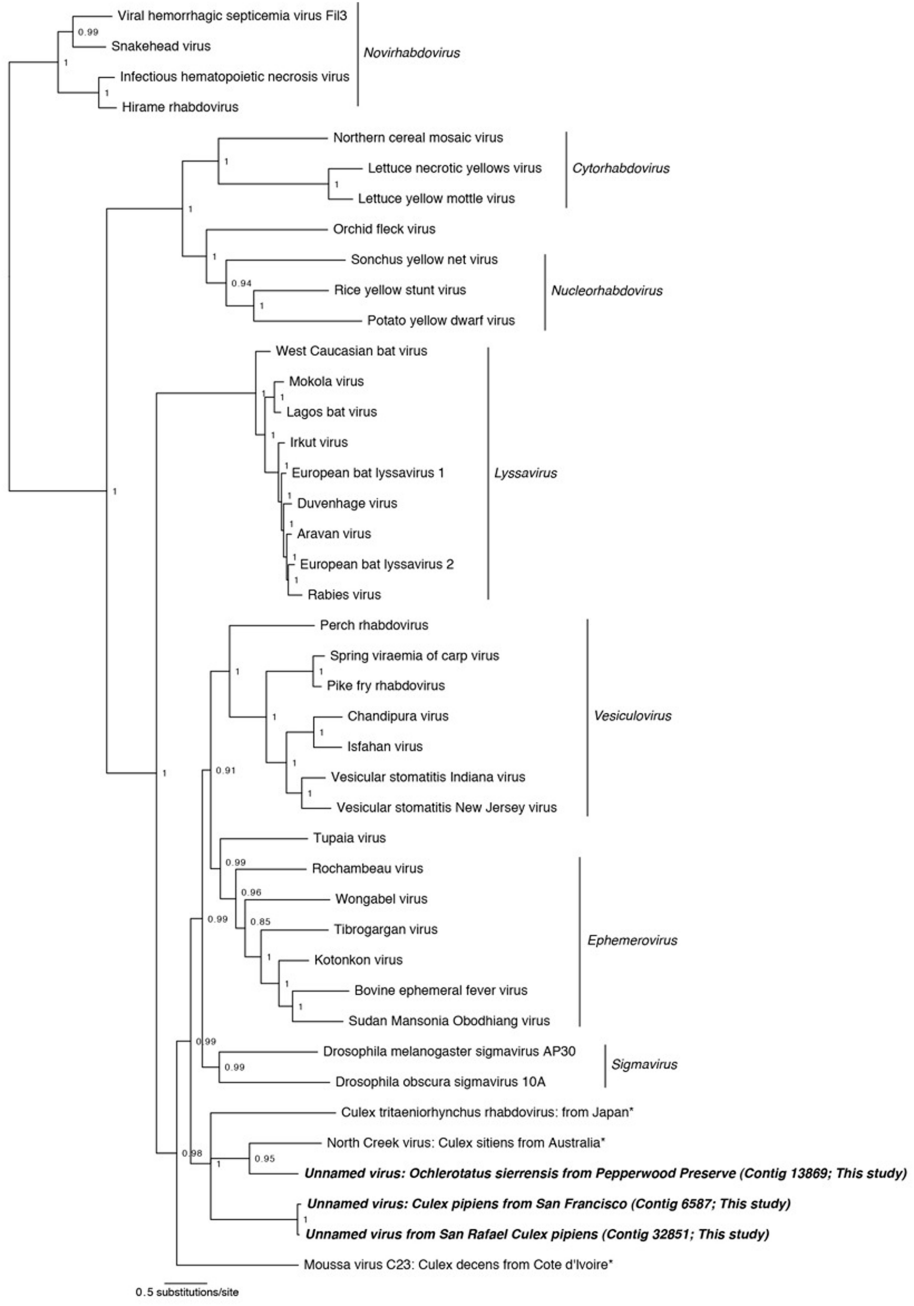
**Phylogenetic history of the *Rhabdoviruses* including sequences from this study.** This consensus phylogeny was generated in MrBayes (Ronquist and Huelsenbeck, 2003) based on two independent chains run for one million generations to convergence. The average standard deviation of split frequencies was 0.002810. Posterior node probabilities are shown at nodes. Branch lengths are scaled to substitutions/site. Viral sequences uncovered in this study are labeled as such. Closely related mosquito-associated viruses are marked with an asterisk. The accession numbers of all sequences are available at http://dx.doi.org/10.6084/m9.figshare.1247641.

**FIGURE 4:**
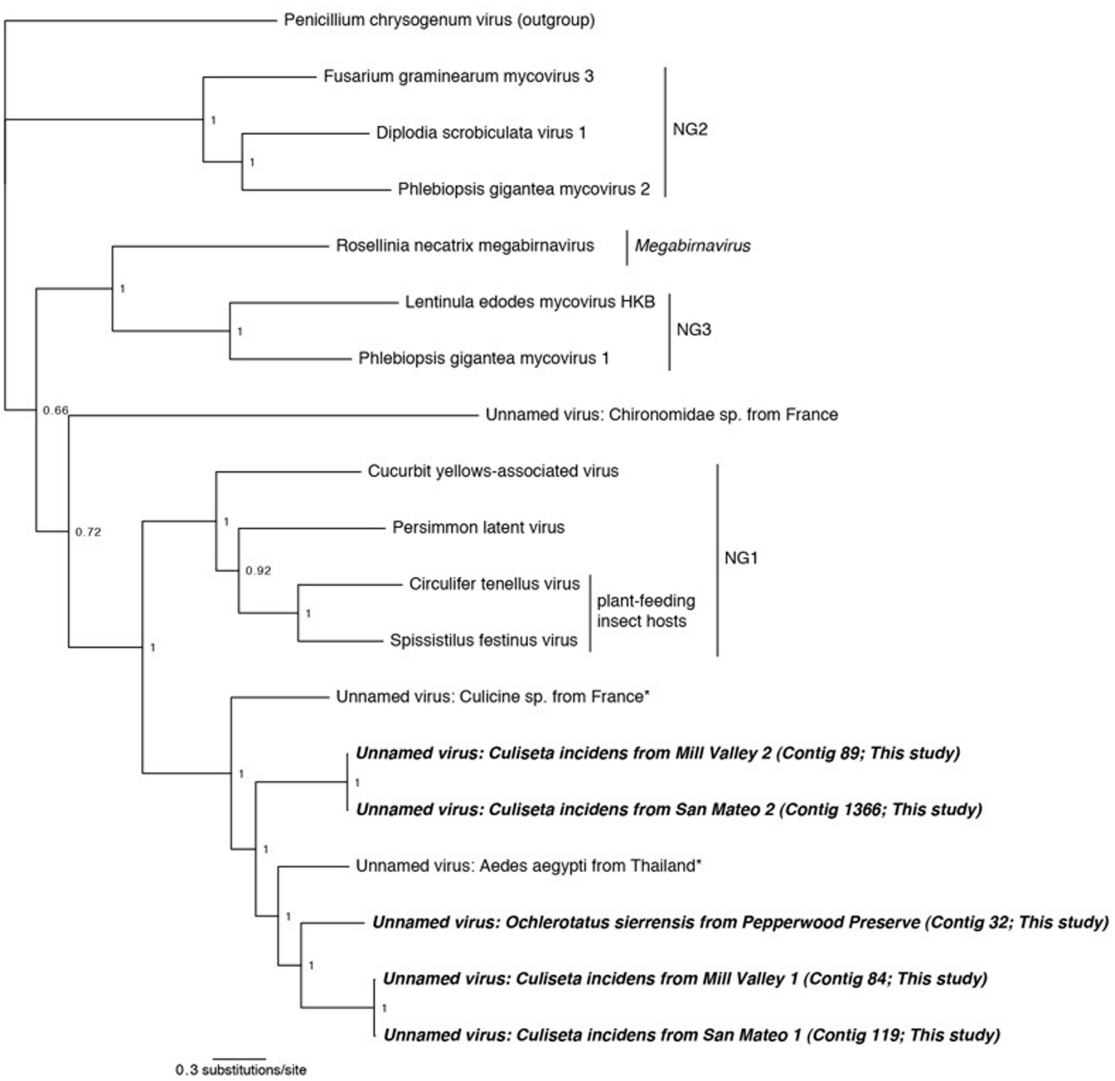
**Phylogenetic history of select dsRNA viruses including sequences from this study and Chandler et al. (2014).** This consensus phylogeny was generated in MrBayes (Ronquist and Huelsenbeck, 2003) based on two independent chains run for one million generations to convergence. The average standard deviation of split frequencies was 0.000948. Posterior node probabilities are shown at nodes. Branch lengths are scaled to substitutions/site. Viral sequences uncovered in this study are labeled as such. Closely related mosquito-associated viruses are marked with an asterisk. As in Ito et al. (2013), *Penicillium chrysogenum* virus is designated the outgroup. Proposed genera (Ito et al., 2013) are designated NG1, NG2, and NG3. The accession numbers of all sequences are available at http://dx.doi.org/10.6084/m9.figshare.1247641.

**FIGURE 5:**
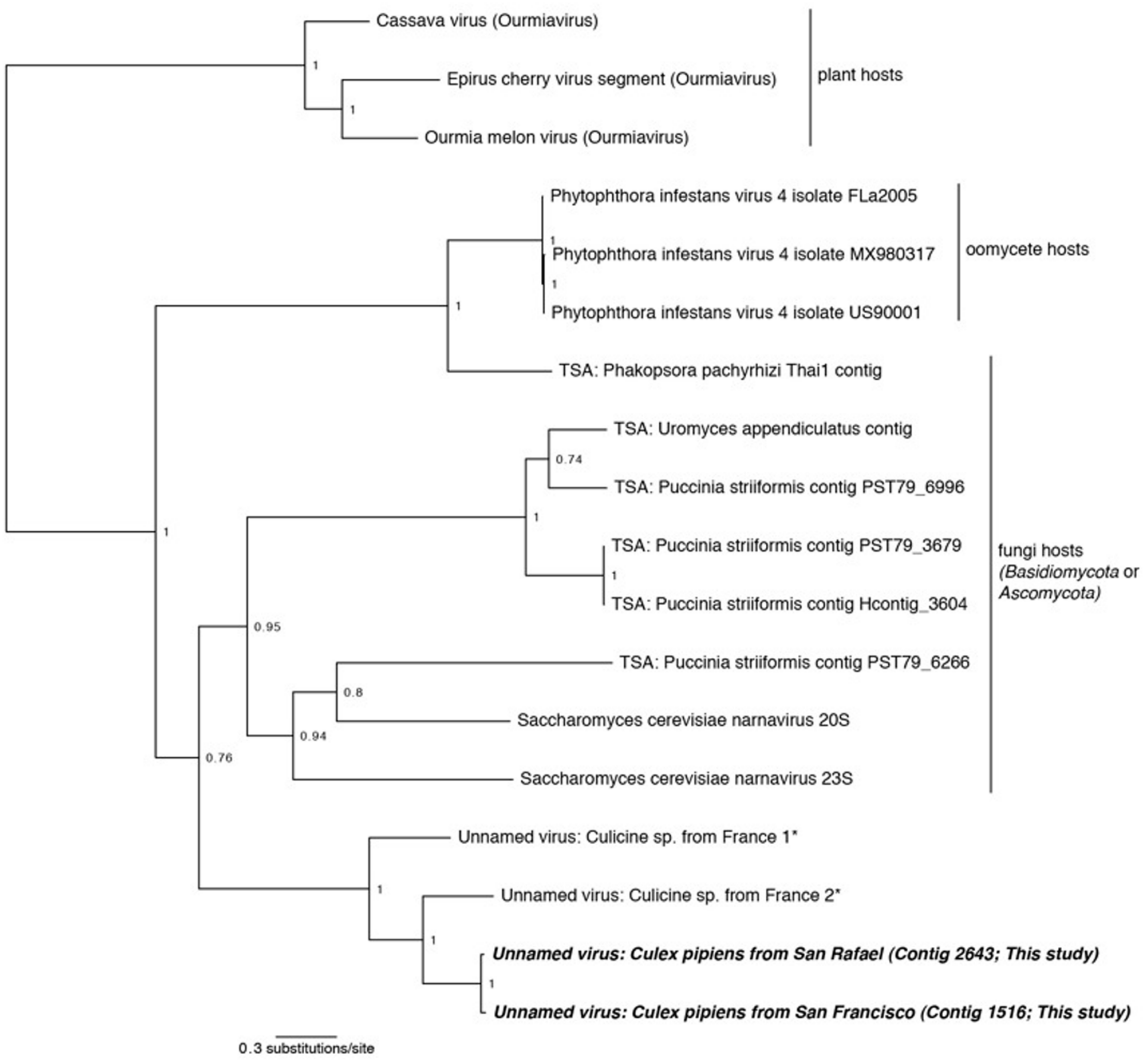
**Phylogenetic history of the *Narnaviruses* including sequences from this study.** This consensus phylogeny was generated in MrBayes (Ronquist and Huelsenbeck, 2003) based on two independent chains run for one million generations to convergence. *Ourmiaviruses,* which share the RNA-dependent RNA polymerase through a genomic rearrangement, are included as the outgroup. The average standard deviation of split frequencies was 0.004048. Posterior node probabilities are shown at nodes. Branch lengths are scaled to substitutions/site. Viral sequences uncovered in this study are labeled as such. Closely related mosquito-associated viruses are marked with an asterisk. Sequences labeled TSA were uncovered in the NCBI Transcriptome Shotgun Assembly database (Cook et al., 2013). The accession numbers of all sequences are available at .

Phylogenetic analysis was performed by comparing the complete and contiguous sequence of each identified viral ORFs to related taxa. Alignments were performed using MAFFT v7.058 and the E-INS-i algorithm (Katoh and Standley, 2013). This alignment algorithm is suitable for sequences that contain multiple conserved regions embedded in long unalignable regions ^2^. Bayesian analysis was performed using MrBayes v3.1.2 (Ronquist and Huelsenbeck, 2003). The substitution model was determined by allowing MrBayes to sample across the fixed amino acid rate matrices. For each ORF, two independent chains were run for 1,000,000 generations. The resulting average standard deviation of split frequencies is indicated in the caption to each figure. Tracer v1.5.0 was used to confirm the stationarity of log likelihoods (Rambaut et al., 2013) and the first 25% of the 10,000 total trees were discarded. Results were visualized using FigTree v1.4.0 (Rambaut, 2012 **Figures 2–5**). All trees are available at . In each case, the alignments contain highly divergent regions without homologs in all taxa, which are encoded with gaps to represent missing data. To confirm these gap sections do not significantly affect reconstruction, we repeated all phylogenetic analyses using only sites that contain data from at least 50% of the taxa. Phylogenetic reconstruction using MrBayes on these shortened alignments found very similar topologies as presented in **Figures 2–5**, and did not change any conclusions (phylogenetic trees resulting from shortened alignments available at http://dx.doi.org/10.6084/m9.figshare.1247641).

Coverage was determined by mapping the quality checked reads to the nucleotide sequences corresponding to the viral ORFs using Bowtie 2 and default settings (Langmead et al., 2009).

## Results and Discussion

In this study, we used RNA SMS to characterize the microbial communities associated with three mosquito species in northern California: *Culex pipiens*, *Culiseta incidens*, and *Ochlerotatus sierrensis* (previously *Aedes sierrensis*). *Cx. pipiens* (common house mosquito) is a known vector for *West Nile virus* and *St. Louis encephalitis virus* (Farajollahi et al., 2011), *C. incidens* (cool-weather mosquito) is not a major vector for *West Nile virus* or any other disease, and *O. sierrensis* (western treehole mosquito) is a major vector for dog heartworm (Ledesma and Harrington, 2011). Samples were collected from a variety of habitats including sylvatic/wild, rural, suburban, and urban (*sensu* Thongsripong et al., 2013). All three of these species have limited ranges (flight range <5 miles), making them good markers for the respective habitats in which they are found. Only female mosquitoes were used, as female mosquitoes, being hematophagous, are the primary vectors of most human and animal diseases.

As many clinically important mosquito-vectored viruses are RNA based (for example, certain *Alphaviruses*, *Bunyaviridae*, and *Flaviviruses*), we focused on the RNA metagenome of these mosquitoes. Nearly 250 million 100 bp paired-end reads were generated on the Illumina HiSeq platform, roughly evenly distributed among the seven libraries (**Table 1**). After quality control and contig assembly, the identity of the viral sequences associated with these samples was determined by querying the translated contigs against a custom database containing the entire NCBI non-redundant protein database, the PhAnToMe phage protein database (Aziz et al., 2012), the NCBI Viral RefSeq Database, and the *Ae. aegypti*, *An. gambiae*, and *Cx. quinquefasciatus* protein databases (Megy et al., 2012). The identity of the bacterial and fungal sequences associated with these samples was determined by first querying the non-translated contigs to the SILVA SSU and LSU Reference Databases (Quast et al., 2013). Any contigs with a closest match to either bacteria or fungi were then submitted to the RDP Classifier (Wang et al., 2007). Despite using Ribo-Zero^TM^ to deplete vertebrate rRNA, adequate bacterial and fungal RNA remained for microbial classification.

### Viral Communities

Consistent with previous studies (Cook et al., 2013; Chandler et al., 2014; Coffey et al., 2014), viral sequences from the lineages *Bunyaviridae*, *Rhabdoviridae*, and *Narnavirus* were uncovered in these mosquitoes (**Table 2**). In addition, we also detected sequences related to an unassigned double-stranded RNA virus (dsRNA) which has been found to be associated with insects (Spear et al., 2010; Cook et al., 2013). No single viral taxon was found in all samples; however, members of the dsRNA group and the *Rhabdoviridae* were detected in three samples. Interestingly, the San Rafael *Cx. pipiens* sample and the San Francisco *Cx. pipiens* sample each contained representatives of the same three viral taxa – *Bunyaviridae*, *Rhabdoviridae*, and *Narnavirus*. Using the methods employed here, viral sequences were not detected in the Stinson Beach or Bolinas *Cx. pipiens* samples. If viruses are present in these samples, they fall below our detection limit (see Section “Materials and Methods”).

**Table 2:** Viral sequences identified in northern California mosquitoes.

Location	Species	Contig designation	Length of ORF^a^	%ID^b^	Viral group^c^	Gene	Depth^d^
Pepperwood Preserve	*O. sierrensis*	32	1011	34.9	dsRNA	RdRp^e^	123
		32	1012	21.4	dsRNA	PArp^f^	123
		13869	151	27.1	*Rhabdoviridae*	Glycoprotein	6
Bolinas	*Cx. pipiens*	none					
Stinson Beach	*Cx. pipiens*	none					
San Rafael	*Cx. pipiens*	30	2435	26.5	*Bunyaviridae*	RdRp	2985
		2643	1006	24.6	*Narnavirus*	RdRp	3815
		32851	101	40.4	*Rhabdoviridae*	Nucleocapsid	3
Mill Valley	*C. incidens*	84	981	32.8	dsRNA	RdRp	45
		84	975	20.9	dsRNA	PArp	45
		89	917	35.4	dsRNA	RdRp	67
		89	1031	23.9	dsRNA	PArp	67
San Francisco	*Cx. pipiens*	84	2371	26.5	*Bunyaviridae*	RdRp	2503
		1516	1027	24.2	*Narnavirus*	RdRp	6199
		6587	408	33.1	*Rhabdoviridae*	Nucleocapsid	31
San Mateo	*C. incidens*	119	981	33.0	dsRNA	RdRp	140
		119	975	20.9	dsRNA	PArp	140
		1366	695	35.5	dsRNA	RdRp	10
		1366	395	23.9	dsRNA	PArp	10
		20718	275	34.5	dsRNA	RdRp	7
		26686	217	28.5	dsRNA	RdRp	6

*^a^In amino acids.*

*^b^To nearest match within the custom blastp database (see Section “Materials and Methods” and supplementary data at http://dx.doi.org/10.6084/m9.figshare.1247641).*

*^c^As determined by nearest blastp match and phylogenetic reconstruction.*

*^d^As determined using by mapping the quality-checked reads back to the assembled contigs using Bowtie 2. Depth is the average coverage across the entire length of the contig. Coverage maps for each contig are available at http://dx.doi.org/10.6084/m9.figshare.1247641*

*^e^RNA-dependent RNA polymerase (RdRp).*

*^f^Proline-alanine rich protein (PArp).*

#### Bunyaviridae

In two samples, *Cx. pipiens* from San Francisco and *Cx. pipiens* from San Rafael, we uncovered sequences related to the *Bunyaviridae* L segment. There are five recognized *Bunyaviridae* genera (*Phlebovirus, Hantavirus*, *Nairovirus*,* Orthobunyavirus*, and *Tospovirus*), and all except *Hantavirus* are known to be arthropod-vectored. The *Bunyaviridae* have single-stranded negative-sense genomes consisting of three segments. In the genus *Phlebovirus* (which our sequences are most closely related to, see next paragraph), these segments are designated L (encoding the RNA-dependent RNA polymerase), M (encoding the glycoprotein), and S (encoding the nucleocapsid and an ambisense non-structural gene, the NSs, which is necessary for virulence in vertebrates).

Phylogenetic analysis indicates that the detected *Bunyaviridae* sequences are sister to the *Phlebovirus* genus, which includes the agent of Rift Valley fever, and relatives such as *Gouléako virus* (**Figure 2**). This is in congruence with several other mosquito-associated *Bunyaviridae* that have been found in Thailand (Chandler et al., 2014), France (Cook et al., 2013), and West Africa (Marklewitz et al., 2011). Although the two uncovered sequences are more closely related to each other than to any other taxa, these two sequences differ by approximately 6 amino acids substitutions and a 64 amino acid insertion/deletion at the terminal end (alignments available at ).

Attempts to find the M and S *Bunyaviridae* segments using specific blastp and vFam searches (Skewes-Cox et al., 2014) were unsuccessful (data not shown). While this is surprising given the depth of coverage of the L segment (**Table 2**), we note that a previous metagenomic shotgun sequencing study which found a *Bunyaviridae* L segment likewise did not find either the M or the S segments (Cook et al., 2013). The inability to recover these segments may reflect their greater degree of divergence than the L segment, or their reduced representation in the libraries. Targeted amplification of the missing segments by PCR could compensate for the latter, but may have limited success in the case of divergence. Previous work finds that some mosquito viruses lack the NSs gene, which is one of two genes, along with the nucleoprotein, present on the S segment (Marklewitz et al., 2011; Chandler et al., 2014). The absence of the NSs, which is found in all *Phleboviruses* and is necessary for virulence in vertebrates, and the presence of the nucleoprotein, which is required for replication, is suggestive of an arthropod-only lifecycle in these viruses (Marklewitz et al., 2011; Chandler et al., 2014).

The nucleic acid contigs of both *Bunyaviridae* detected here included stop codons on either side of the ORF suggesting that the entire ORF was found. However, the terminal hairpins that are present in all Bunyaviridae were not recovered, despite implementing PRICE (Ruby et al., 2013) to extend the fragments (data not shown). This suggests that the entire L segment was not present in our data.

#### Rhabdoviridae

In three of our samples, *O. sierrensis* from Pepperwood Preserve, *Cx. pipiens* from San Rafael, and *Cx. pipiens* from San Francisco, we detected sequences related to the family *Rhabdoviridae*. The *Rhabdoviridae* is a diverse family in the order *Mononegavirales* and consists of six established genera (*Vesiculovirus, Lyssavirus, Ephemerovirus, Novirhabdovirus, Cytorhabdovirus,* and *Nucleorhabdovirus*) along with more than 130 unassigned viruses, such as the *Drosophila*-associated *Sigma viruses* (Kuzmin et al., 2009; Longdon et al., 2010). *Rhabdoviridae* are the causative agents of numerous clinically and economically important diseases of humans, livestock, fish, and plants – some of which are arthropod-vectored (Kuzmin et al., 2009). The genome of the *Rhabdoviridae* consists of a single negative-sense RNA segment with, at minimum, five ORFs corresponding to the nucleoprotein, glycoprotein, phosphoprotein, matrix protein, and the RdRp.

Using our methods, we uncovered partial fragments of the *Rhabdoviridae* nucleoprotein (*Cx. pipiens* from San Rafael and *Cx. pipiens* from San Francisco) and the *Rhabdoviridae* glycoprotein (*O. sierrensis* from Pepperwood Preserve). Since the RdRp is the preferred gene for phylogenetic reconstruction (Bourhy et al., 2005; Longdon et al., 2010), we searched for it within these three libraries using specific blastp and vFam searches (Skewes-Cox et al., 2014), but did not recover the RdRp in our datasets. This is unsurprising since the depth of coverage for the three uncovered fragments was very low compared to many of the other uncovered viruses (**Table 2**).

Phylogenetic analysis was performed using MrBayes on concatenated alignments of the nucleoprotein and glycoprotein fragments against reference sequences for these regions (**Figure 3**). The resulting phylogeny successfully recapitulates the relationships between *Rhabdoviridae* lineages ((((*Vesiculovirus, Ephemerovirus), Sigma viruses*), *Lyssavirus), (Cytorhabdovirus, Nucleorhabdovirus*), *Novirhabdovirus*) found by other studies (Longdon et al., 2010; Coffey et al., 2014). Our mosquito-associated *Rhabdoviridae* form a clade with *North Creek virus*, isolated from *Cx. sitiens* in Australia (Coffey et al., 2014) and a unnamed virus isolated from *Cx. tritaeniorhynchus* in Japan (Kuwata et al., 2011). This mosquito-associated clade is sister to the *Vesiculovirus, Ephemerovirus,* and* Sigma virus* clades; basal to these is the mosquito-associated *Moussa virus* (Quan et al., 2010).

#### Double-Stranded RNA Viruses

In three libraries, we identified sequences that are related to dsRNA viruses previously identified in culicine mosquitoes from France (Cook et al., 2013). These viruses are related to, and have a similar genome structure as, viruses isolated from the plant-feeding insects *Circulifer tenellus* and *Spissistilus festinus* (Spear et al., 2010). The genomes of these viruses consist of a single segment with two ORFs, an RNA-dependent RNA polymerase (RdRp) and a proline–alanine rich protein (PArp) of unknown function. Phylogenetic analysis of the RdRp suggests that the viruses from plant-feeding insects belong to a novel genus of dsRNA viruses (Spear et al., 2010). Phylogenetic analysis using the PArp ORF has not been performed in any previous publication.

We identified sequences related to these dsRNA viruses in three of our samples: *O. sierrensis* from Pepperwood Preserve, *C. incidens* from Mill Valley, and *C. incidens* from San Mateo. Inspired by the discovery of these viral sequences, we searched a prior dataset from our laboratory [*Ae. aegypti* from Thailand (Chandler et al., 2014)] using the methods described above and identified another dsRNA virus. In several instances, a single contig spanned the entire RdRp and PArp ORFs (contig 32 in the *O. sierrensis* sample from Pepperwood Preserve, contigs 84 and 89 in the *C. incidens* sample from Mill Valley, and contig 119 in the *C. incidens* sample from San Mateo; **Table 2**). In the San Mateo sample, one low coverage contig contains partial sequences of both the RdRp and the PArp (contig 1366) and two additional low coverage contigs (contigs 20718 and 26686) contain fragments of the RdRp that partially overlap with, and are nearly identical to, the contig containing the partial sequences.

Phylogenetic analysis was performed using the complete RdRp ORFs and the partial ORF from the San Mateo sample (contig 1366). Viruses from three proposed novel genera, one *Megabirnavirus*, and *Penicillium chrysogenum* virus were included as comparison taxa (Spear et al., 2010; Ito et al., 2013). Our phylogeny reproduces the overall structure of previous work (Ito et al., 2013) and places all mosquito-associated viruses together (**Figure 4**). Interestingly, both the San Mateo and Mill Valley samples have two distinct dsRNA contigs. This suggests that multiple strains of this dsRNA virus are co-circulating within the northern California *C. incidens* population, a situation similar to that occurring in *Circulifer tenellus* in central California (Spear et al., 2013).

In the dsRNA plant-feeding insect viruses, the RdRp and PArp ORFs are not in frame, but are suspected to be transcribed as a fusion via a -1 ribosomal frameshift just prior to the stop codon of the 5′ (PArp) ORF. The site of this frameshift is predicted to occur at a G_GAA_AAC_stop motif in the virus of French culicine mosquitoes (Cook et al., 2013). In all of our contigs, the PArp and the RdRp were a single base out of frame. In three of our contigs (84 from Mill Valley *C. incidens*, 32 from Pepperwood Preserve *Cx. pipiens*, and 119 from San Mateo *C. incidens*), we identified a G_GAA_AAC_stop motif and in the remaining contigs (89 from Mill Valley *C. incidens* and 1366 from San Mateo *C. incidens*), we identified a G_GGA_AAC_stop motif. This suggests that the two dsRNA ORFs found here are transcribed as a fusion, although enabled by slightly different motifs.

#### Narnavirus

Two of our samples, *Cx. pipiens* from San Rafael and *Cx. pipiens* from San Francisco, included sequences that are related to the genus *Narnavirus* in the family *Narnaviridae*. *Narnaviruses* have single-stranded positive-sense genomes consisting of a single ORF that encodes an RdRp (King et al., 2012). The type strain *Saccharomyces 20S virus* infects the Ascomycota *Saccharomyces cerevisiae*, while similar sequences have been found associated with Basidiomycota fungi (Cook et al., 2013) and an oomycete plant pathogen (Cai et al., 2013). While the sister genus to *Narnavirus* is the mitochondrial-infecting *Mitovirus*, the RdRp of plant-infecting *Ourmiavirus* is more closely related to *Narnavirus*, as it was acquired via reassortment (Rastgou et al., 2009), and is therefore used as the outgroup in **Figure 5**.

Phylogenetic analysis finds that the *Cx. pipiens Narnaviruses* are more closely related to the other mosquito-associated *Narnaviruses* (Cook et al., 2013) than to any other taxa (**Figure 5**). Since most of the known *Narnaviruses* are suspected to infect Ascomycete or Basdiocymete fungi [the only exception being the oomycete-infecting *Phytophthora infestans RNA virus* (Cai et al., 2013)], we investigated the presence and identity of potential host fungal sequences in our samples (see Section “Fungal Communities”). We also used the methods described in Section “Sequence Processing and Taxonomy Assignment” to identify fungal sequences in the French mosquito dataset that contained two *Narnavirus* sequences (Cook et al., 2013). We did not find any oomycete SSU or LSU sequences in any of the three datasets (data not shown), thus ruling out that the mosquito-associated *Narnaviruses* are in fact associated with the presence of oomycetes.

All three mosquito samples positive for *Narnaviruses* also included sequences of Ascomycete fungi. Although no single fungal genus was common to all three mosquito samples, several [such as *Cladosporium* and *Capnodiales* (see Section “Fungal Communities”)] were shared between the two northern California samples. Considering that the mosquito-associated *Narnavirus* clade is sister to *Narnaviruses* of fungi, and that fungal sequences were also detected in the mosquitoes, the *Narnaviruses* from mosquitoes reported here and by Cook et al. (2013) may in fact be instances of virus-infected fungal infections of mosquitoes. Alternatively, these mosquito-associated *Narnaviruses* may represent direct infections of mosquitoes. However, *Saccharomyces 20S RNA virus* is vertically transmitted from mother to daughter cells and horizontally through mating (King et al., 2012), suggesting that host switching may be biologically improbable, particularly from a fungal host to a mosquito host. Taken together, our approach may have revealed an interesting scenario: since the method employed here simultaneously characterized both viruses and fungi within a given sample, it can identify patterns of multi-level nested host-parasite associations. In this case, we have identified putative fungal infecting viruses and the fungi they may be infecting, all in the same hosts. This illustrates the potential utility of our broad approach to microbiota characterization – one that could reveal microbial interactions that may ultimately lead to the development of infectious disease control measures.

#### Bacteriophages

No phage sequences were uncovered in this dataset, despite including the PhAnToMe phage database in our blastp search. Given that these mosquitoes were associated with a diversity of bacteria (see Section “Bacterial Communities”) and that the microbiota of other animals consists of abundant phage (De Paepe et al., 2014), it is reasonable to presume that phages are indeed associated with these mosquitoes as well. There exist two explanations for our inability to find phage associated with these samples. First, our dataset was RNA based and most phages are DNA based. Indeed, known phages of the two most widespread bacteria associated with these samples (*Bacillus* and *Escherichia/Shigella*; see Section “Bacterial Communities”) are double-stranded DNA based. Second, perhaps the phages infecting the bacteria within these mosquitoes are undescribed and therefore would not be represented in the databases.

### Bacterial Communities

Contigs that could be confidently identified as bacteria using the RDP Classifier were found in all seven samples (**Table 3**). Since there are known limitations of using rRNA as a measurement of either microbial abundance or activity (Blazewicz et al., 2013), the quality-checked reads were not mapped back to these contigs to determine the number of reads used to build each contig. Therefore the data presented here (and of the fungi discussed below) should only be interpreted as representing the presence of that particular taxon and not that taxon’s abundance within the community.

**Table 3:** Bacterial sequences associated with northern California mosquitoes.

Location	Species	Contig designation	Contig length	Family	F %^a^	Genus	G %^b^
Pepperwood preserve	*O. sierrensis*	1977	1546	Bacillaceae 1	100	*Bacillus*	100
		18821	401	Corynebacteriaceae	100	*Corynebacterium*	100
		1951	1521	Enterobacteriaceae	100	*Escherichia/Shigella*	100
Bolinas	*Cx. pipiens*	9384	632	Pasteurellaceae	100	*Actinobacillus*	93
		12068	527	Anaplasmataceae^c,d^	78	*Anaplasma*^c,d^	76
		20316	353		46		36
		12181	534		26		17
		13969	492	Bacillaceae 1^d^	100	*Bacillus*^d^	100
		18774	409		98		98
		20021	394	Enterobacteriaceae^d^	100	*Escherichia/Shigella*^d^	100
		16291	447		100		99
Stinson Beach	*Cx. pipiens*	2161	1446	Anaplasmataceae^c^	99	*Anaplasma*^c^	97
		2025	1539	Bacillaceae 1	100	*Bacillus*	100
		1856	1515	Enterobacteriaceae	100	*Escherichia/Shigella*	100
San Rafael	*Cx. pipiens*	4009	1373	Anaplasmataceae^c^	91	*Anaplasma*^c^	90
		4901	333^e^	Bacillaceae 1	100	*Bacillus*	100
		29993	328	Enterobacteriaceae	100	*Escherichia/Shigella*	99
Mill Valley	*C. incidens*	9233	365	Bacillaceae 1	100	*Bacillus*	100
		7981	658	Enterobacteriaceae	100	*Escherichia/Shigella*	100
		7622	713	Moraxellaceae^d^	100	*Moraxella^d^*	100
		8692	521		100		100
		11314	369		100		100
		10818	394	Propionibacteriaceae	100	*Propionibacterium*	100
San Francisco	*Cx. pipiens*	6804	1445	Anaplasmataceae^c^	99	*Anaplasma*^c^	98
		5560	1545	Bacillaceae 1	100	*Bacillus*	100
		16616	579	Enterobacteriaceae	100	*Escherichia/Shigella*	98
		12354	936	Moraxellaceae	100	*Moraxella*	100
San Mateo	*C. incidens*	8563	1498	Bacillaceae 1	100	*Bacillus*	100
		8084	1521	Enterobacteriaceae	100	*Escherichia/Shigella*	100
		24207	781	Pseudomonadaceae	100	*Pseudomonas*	100


^a^RDP Family level confidence.

^b^RDP Genus level confidence.

^c^Manually confirmed to be *Wolbachia* by querying to the NCBI non-redundant database.

^d^Multiple contigs from the same sample match to the same bacterial genus.

^e^Only the bacterial portion of this chimeric contig was used.

Contigs (those that passed the initial 300 bp cutoff) ranged from 328 to 1546 bases in length, which means that some cover nearly the entire 16S gene and all cover at least one hyper-variable region. A single contig with a bacterial match (*Bacillus* in the Mill Valley *C. incidens* sample) was found to be chimeric (data not shown) and only the bacterial portion was used for classification. We identified the following bacterial genera in our samples:* Actinobacillus, Anaplasma* [*Wolbachia* (see below)], *Bacillus, Corynebacterium, Escherichia/Shigella, Haemophilus, Moraxella, Propionibacterium, Pseudomonas* and *Wandonia*. When a genus was found multiple times in the same sample (for example, *Moraxella* in the Mill Valley *C. incidens* sample), the contigs spanned different regions of the 16S gene (see blastn results at ).

*Bacillus* and *Escherichia/Shigella* were the most widespread genera, as they were found in all seven samples. Notably, a recent review by Minard et al. (2013) concludes that female mosquitoes are mostly colonized by Gammaproteobacteria (of which *Escherichia/Shigella* is a member) and that males are dominated by Firmicutes, such as *Bacillus*. The results of our study, in which only females were investigated, are therefore in partial agreement with this conclusion.

*Anaplasma* is a genus in the order Rickettsiales. *Wolbachia* is a notable and widespread arthropod-associated bacteria in this order (Hilgenboecker et al., 2008) and we therefore investigated the *Anaplasma* contigs further. Querying these contigs to the NCBI database finds them to be greater than 98% identical to *Wolbachia pipiens* (data not shown). The two samples containing *Wolbachia* were *Cx. pipiens* from San Rafael and Stinson beach. The third *Cx. pipiens* sample, from Bolinas, also had contigs identified as *Anaplasma*, although their confidence was below the 90% cutoff in RDP (**Table 3**). Querying these contigs to NCBI found they were greater than 99% identical to *Wolbachia pipiens* (data not shown, all contigs are available at ). Inspection of the contigs from *C. incidens* and *O. sierrensis* that were shorter than 300 bp or below the 90% confidence cutoff did not reveal any *Anaplasma* or *Wolbachia*-like sequences.

*Wolbachia* was first described in *Cx. pipiens* mosquitoes (Hertig and Wolbach, 1924). A survey of California mosquitoes (including *Culex, C. incidens,* and *Ochlerotatus*) for *Wolbachia* infections found that, out of 296 individuals and 14 species, only *Cx. pipiens* mosquitoes were infected (Rasgon and Scott, 2004). How *Wolbachia*, which is vertically transmitted, spreads into new taxa is not well understood, but its absence in certain species could be due to inhibition by other members of the mosquito microbiota, as has been shown in *Anopheles* mosquitoes (Hughes et al., 2014). While this study did not find any consistent microbiota differences between *Wolbachia*-infected and *Wolbachia*-free mosquitoes, more comprehensive studies may find such differences that might help account for natural *Wolbachia* distributions.

Of final note, two of the bacterial genera identified, *Propionibacterium* and *Corynebacterium,* are commonly associated with human skin (Grice and Segre, 2011). There are two potential explanations for their presence in these samples. One is that they were acquired from human hosts during a blood meal. Alternatively, these could be contaminants introduced during collection or processing for sequencing. Future studies will include a negative control to identify this possibility (Salter et al., 2014).

### Fungal Communities

Contigs that could be confidently classified as fungi using the RDP Classifier were found in all samples except *O. sierrensis* from Pepperwood Preserve (**Table 4**). Manually querying the putative fungal contigs (identified by the initial SILVA database search) from this sample against the NCBI database confidently identified a fungus that was nonetheless below the RDP confidence cutoff (**Table 4**).

**Table 4:** Fungal sequences associated with northern California mosquitoes.

Location	Species	Contig designation	Contig length	Family	F %^a^	Genus	G %^b^
Pepperwood Preserve	*O. sierrensis*	15039	460	Ascomycota incertae sedisa^c^	21	*Capnobotryella*	19
Bolinas	*Cx. pipiens*	12631	512	Trichocomaceae	89	*Chromocleista*	63
Stinson Beach	*Cx. pipiens*	13384	473	Trichocomaceae	87	*Aspergillus*	48
		26216	322	Trichocomaceae	88	*Chromocleista*	35
		23528	346	Trichocomaceae	99	*Eurotium*	94
		20005	332	Exobasidiaceae	100	*Exobasidium*	100
		19659	379	Cystofilobasidiaceae^d^	100	*Guehomyces*^d^	100
		13424	302		100		100
		20700	376	Clavulinaceae^d^	100	*Multiclavula*^d^	100
		21025	372		75		61
		6619	772	Tricholomataceae^d^	86	*Mycenella*^d^	86
		7662	701		96		96
		3091	1173	Coriolaceae	100	*Poria*	100
San Rafael	*Cx. pipiens*	13553	673	Davidiellaceae	100	*Cladosporium* complex	83
Mill Valley	*C. incidens*	10362	337	Erysiphaceae	98	*Arthrocladiella*	72
San Francisco	*Cx. pipiens*	24609	548	Mycosphaerellaceae	100	*Cercosporella*	87
		23638	565	Davidiellaceae	100	*Cladosporium* complex	63
		52139	304	Clavicipitaceae	100	*Claviceps*	100
		12695	931	Malasseziaceae	100	*Malassezia*	100
		50818	310	Saccharomycetaceae	100	*Saccharomyces*	100
San Mateo	*C. incidens*	86913	332	Trichocomaceae	74	*Eupenicillium*	44
		61855	433	Helotiales incertae sedis	86	*Tetracladium*	86

^a^RDP Family level confidence.

^b^RDP Genus level confidence.

^c^Manually confirmed to belong to the family Cordycipitaceae by querying to the NCBI non-redundant database.

^d^Multiple contigs from the same sample match to the same fungal genus.

Fungal contigs ranged in length from 302 to 1173 bp. Fungi from 13 families were present in our data (**Table 4**). In contrast to the bacterial data, there is no single fungal genus that is present in all samples and only two fungi were found in multiple samples. Specifically, *Chromocleista* was found in *Cx. pipiens* from both Stinson Beach and Bolinas, and *Cladosporium* was found in *Cx. pipiens* from both San Rafael and San Francisco. All the remaining fungal taxa identified were unique to a single sample. More in-depth sequencing may reveal widespread fungi that exist at numbers within the host too low for our methods to detect.

Some of the fungi identified within these samples may not exist in an intimate relationship with mosquitoes. For example, several taxa within the Basidiomycota have a characteristic multicellular stage, such as the *Mycenella* gilled mushrooms identified in the *Cx. pipiens* sample from Stinson Beach. Most likely, dispersing spores of these fungi contaminated water sources used by adult mosquitoes as breeding sites. Similarly, the fungal taxa *Malassezia*, identified in *Cx. pipiens* from San Francisco, is a common human commensal and opportunistic pathogen (Gupta et al., 2004) that likely contaminated the sample through human contact, as with the bacteria discussed above.

Much previous work has focused on using entomopathogenic fungal taxa for mosquito control (Scholte et al., 2004; de Faria and Wraight, 2007). Interestingly, several of the fungi identified belong to families that include many known entomopathogenic fungi including Clavicipitaceae (found in the *Cx. pipiens* sample from San Francisco) and Cordycipitaceae (found in the *O. sierrensis* sample from Pepperwood Preserve). For future mosquito collections, histologic examination would be necessary to identify visible signs of disease in positive mosquitoes.

This study represents, to our knowledge, the first to use culture-independent techniques to characterize fungal communities in natural populations of mosquitoes. As with other animal hosts [e.g., humans (Huffnagle and Noverr, 2013) and *Drosophila* (Broderick and Lemaitre, 2012)], fungi associated with mosquitoes are relatively understudied compared to bacteria. One study of laboratory-raised *Ae. aegypti* identified *Saccharomyces, Penicillium,* and *Aspergillus* using methods similar to those employed here (Bishop-Lilly et al., 2010). While the current study did find these, or closely related, taxa, more work is needed to fully determine how the fungal communities of laboratory-raised and wild-caught mosquitoes compare.

### Mosquito Identification

Five of the seven mosquitoes were identified morphologically or by PCR amplification and Sanger sequencing of the COI gene prior to SMS. For the two specimens that were not identified beforehand, contigs matching mosquito COI and mosquito LSU and SSU were queried against the NCBI database and indicated that these mosquitoes were *Cx. pipiens* and *O. sierrensis* (data not shown, all contigs available at ).

## Conclusion

In this study, we used SMS to identify viral, bacterial, and fungal sequences associated with naturally collected mosquitoes. Notably, in all three cases of mosquito-associated viruses (i.e., the *Bunyaviridae*, *Rhabdoviridae*, and dsRNA viruses) the viruses described here were more closely related to other mosquito-associated viruses than to any other viruses. This is despite the fact that the mosquitoes belonged to a variety of taxa (e.g., *Ae. aegypti, C. incidens, O. sierrensis,* and several species of *Culex*) and were collected from a variety of locations (e.g., northern California, Thailand, France, western Africa, and Australia). Furthermore, it should be noted that two of the viral groups discussed here (*Bunyaviridae* and *Rhabdoviridae*) have been extensively studied due to their importance in human and animal health and are therefore well characterized in terms of host breadth. Taken together, a pattern of family level host-specificity (i.e., to the family Culicidae) is emerging. We look forward to future research to sample the viral taxa more extensively and help answer the question of host-specificity and host-switching in the viral tree of life.

With respect to the bacterial and fungal components of the mosquito microbiota, two opposing patterns emerged: several bacterial taxa were widespread regardless of mosquito species or habitat, whereas fungal communities were distinct between individuals even within host species or habitat. These patterns may be an artifact of insufficient sample size and sequencing depth. Much of our sequencing effort was needlessly expended on host rRNA, as over a quarter of the total data can be attributed to the mosquito ribosomal large subunit or small subunit. However, recent techniques have been developed to selectively remove sample-specific rRNA from environmental (Stewart et al., 2010) and mosquito (Kukutla et al., 2013) samples. The use of these techniques will greatly increase the power to detect bacteria, fungi, and viruses in field-caught mosquitoes. Furthermore, many more samples could be combined into a single experiment to allow for the inclusion of multiple replicates per population, host species, or habitat type. Additionally, the physical environment of the mosquitoes, such as larval breeding sites and attractants used in collection, can be sampled. In this way, the complex interactions between host, environment, and all the microbes inhabiting both, can be determined.

## Confilct of interest statement

The authors declare that the research was conducted in the absence of any commercial or financial relationships that could be construed as a potential conflict of interest.

## Supplementary material

The supplemental data associated with this work is available at: http://dx.doi.org/10.6084/m9.figshare.1247641. This includes alignments, tree files, blast outputs, and other intermediate files.

## Footnotes

1 https://github.com/jts/sga/tree/master/src/

2 http://mafft.cbrc.jp/alignment/software/algorithms/algorithms.html
